# The coverage of SARS-CoV-2 vaccination and the willingness to receive the SARS-CoV-2 variant vaccine among employees in China

**DOI:** 10.1186/s12889-023-15294-7

**Published:** 2023-03-22

**Authors:** Xi-Ru Zhang, Zhi-Ju Li, Qi Fu, Jin-Dong Wang, Qing-Mei Huang, Wei-Qi Song, Xiao-Yu Xu, Zhi-Hao Li, Chen Mao

**Affiliations:** 1 Department of Epidemiology, School of Public Health, Southern Medical University, Guangzhou, Guangdong China; 2 Microbiome Medicine Center, Department of Laboratory Medicine, Zhujiang Hospital, Southern Medical University, Guangzhou, China

**Keywords:** Coronavirus disease 2019, Vaccination, Coverage, Willingness, Related factor

## Abstract

**Background:**

COVID-19, which is caused by SARS-CoV-2, is a major global health threat. The dominant variant of SARS-CoV-2 has changed over time due to continuous evolution. We aimed to evaluate the coverage of SARS-CoV-2 vaccination among employees in China, explore their willingness to receive the SARS-CoV-2 variant vaccine and examine the potential factors influencing vaccination coverage and willingness.

**Methods:**

A cross-sectional epidemiological survey was conducted online from January 1, 2022, to January 30, 2022. The information collected in the survey included sociodemographic characteristics, lifestyle habits, vaccination coverage, willingness to be vaccinated against SARS-CoV-2 variants and the reasons for vaccination and willingness. Multivariable logistic regression models were used to assess the associations of potential factors with the rate of vaccination and the willingness to be vaccinated.

**Results:**

Among 62,395 eligible participants, the coverage of SARS-CoV-2 vaccination was 98.9% for at least one dose and 70.1% for a booster. The great majority of vaccinated individuals (94.4%) voluntarily received the vaccine. A total of 60,694 respondents (97.7%) were willing to be vaccinated against SARS-CoV-2 variants, mainly due to confidence in the effectiveness of vaccines (92.8%). A total of 1431 respondents were unwilling to be vaccinated, mainly because of concerns about the adverse effects of vaccines (77.6%). Longer education duration was associated with a higher rate of SARS-CoV-2 vaccination and willingness to be vaccinated. General or poor health status and having no history of influenza vaccination were associated with a lower rate of SARS-CoV-2 vaccination and willingness to be vaccinated. Additionally, we observed a significant positive association of abuse experience with the willingness to be vaccinated.

**Conclusion:**

Although the rate of SARS-CoV-2 vaccination and the willingness to be vaccinated were relatively high in the study population, there were still some respondents with vaccine hesitancy. Relevant strategies based on significant related factors should be developed and implemented to encourage vaccination.

**Supplementary Information:**

The online version contains supplementary material available at 10.1186/s12889-023-15294-7.

## Introduction

The COVID-19 pandemic has disrupted many aspects of life worldwide and continues to pose a great public health concern. As of February 1, 2022, over 375 million confirmed cases of COVID-19 and 5.6 million deaths, have been reported globally [1]. COVID-19 is caused by SARS-CoV-2, a positive-sense single-stranded RNA virus, which has a higher mutation rate than DNA viruses [2, 3]. Several SARS-CoV-2 variants of concern, with evidence of increased virulence and transmissibility, along with changes to antigenicity, have emerged since late 2020 [3–7]. Delta variants first emerged in India in October 2020, spread rapidly and dominated the strains globally in the second half of 2021 [5]. Omicron variants were first detected in multiple countries in November 2021 and have gradually become the most prevalent variants [4, 6, 7].

Vaccination is a remarkably effective and efficient measure to control COVID-19 and prevent severe illness and hospitalization. With changing antigenicity, notable variants, such as Delta [8–10] and Omicron [7, 11–13], might allow the virus to escape the present vaccine and antibodies produced by it [14]. Therefore, offering booster doses of the SARS-CoV-2 vaccine and developing vaccines targeting emerging variants are urgently needed [15]. Some previous studies have assessed the coverage and acceptance of SARS-CoV-2 vaccines [16]. However, the coverage might have changed with the vaccination process, and the acceptance of future vaccination might be affected by prior SARS-CoV-2 infection, a successful vaccination experience, and even social media.

In this study, we evaluated the current coverage of SARS-CoV-2 vaccination and individuals’ willingness to receive the SARS-CoV-2 variant vaccine and the reasons for both among Chinese employees. Further, we explored the potential factors influencing vaccination coverage and willingness, aiming to formulate and implement relevant strategies to improve acceptance of vaccination and compliance, whether now or in the future.

## Methods

### Study setting and participants

A cross-sectional epidemiological online survey was conducted in a large labour-intensive group in Shenzhen from January 1, 2022, to January 30, 2022, which has more than 150,000 employees from 34 provinces or regions across China. The inclusion criteria were as follows: (1) aged 18-60 years, (2) not blind, (3) employment duration of more than 3 months, and (4) in-service staff of the group. Half of the employees in each position were randomly selected and asked to complete a self-report, online questionnaire, which took approximately 8-15 min to complete. Each IP address was allowed one-time access to the questionnaire to ensure that each subject submitted only one questionnaire. Additionally, an automated logical check function was set up for this questionnaire, and participants were asked to check and amend the option when a logical error was identified. Thus, 62,395 respondents who voluntarily completed the survey were included for an 83.2% effective response rate. The distribution of the ultimately enrolled participants from various provinces or regions is shown in the Supplement (Table S1). The collected information was as follows: sociodemographic characteristics, health status, lifestyle habits, coverage of SARS-CoV-2 vaccination and reasons, and willingness to receive the SARS-CoV-2 variant vaccine and reasons. The protocol of this study was approved by the Institutional Ethics Committee of Zhujiang Hospital of Southern Medical University. All methods were carried out in accordance with relevant guidelines. Informed consent was obtained from all the respondents prior to the beginning of the survey.

### Definitions of independent variables

The sociodemographic characteristics, health status, and lifestyle habits were defined as follows: sex (male or female), age group (18-24, 25-34, 35-44, or 45-60 years), education duration (1-6, 7-9, 10-12, or 13-22 years), ethnicity (Han or minorities), residence (urban or rural), marital status (married, unmarried, divorced or others), health status (very healthy, well, general or poor), position in the group (general worker, line supervisor, group leader, or manager), and history of influenza vaccination before the COVID-19 pandemic (yes or no). Health status was self-reported via an electronic questionnaire. Participants were asked “How do you feel about your health status?” The options for responses were “healthy”, “well” and “general or poor”. An operator on the production line or administrative staff at the lowest position was defined as a general worker. The line supervisor is responsible for the production, monitoring and quality control of a specific production line, while the group leader is responsible for several production lines. The administrative staff in higher positions, such as a section chief, a special manager, a director, a president, or an engineer, were defined as managers. Participants were asked, “Have you experienced abuse in the past six months?” They could choose one of the provided responses. According to abuse experience, participants were divided into four groups: none, verbal abuse, physical abuse (assault, battery, injury, etc.), or both verbal and physical abuse.

### Assessment of the SARS-CoV-2 vaccination coverage as well as the reasons for vaccination

Single-dose or two-dose SARS-CoV-2 vaccines were available in the primary vaccination series. The single-dose vaccine is the recombinant adenovirus vaccine developed by CanSino, and the two-dose vaccine is the inactivated vaccine produced by Sinopharm, Sinovac, and Wuhan Biotech. The full primary vaccination series was defined as receiving the second dose of the two-dose vaccine or the single-dose vaccine. Subsequently, participants could receive a booster. The coverage of SARS-CoV-2 vaccination was classified as “Unvaccinated”, “Only having received the first dose of the two-dose vaccine”, “Having fully received the second dose of the two-dose vaccine”, “Having fully received the single-dose vaccine”, and “Having received the booster dose of SARS-CoV-2 vaccine after completing the primary vaccination series”. A person who received any dose of SARS-CoV-2 vaccine was defined as a “vaccinated individual”.

All vaccinated individuals were asked, “Why did you receive the SARS-CoV-2 vaccine?” Respondents could choose one or more of the following four options: “I voluntarily received the vaccine”, “The managers of the groups or relevant authorities required me to receive the vaccine”, “My relatives or friends encouraged me to receive the vaccine”, and “I was worried that people around me might be prejudiced against me if I refused to get the vaccine”.

### Assessment of the willingness to receive the SARS-CoV-2 variant vaccine as well as the reasons

Each participant was asked, “Would you be willing to receive the SARS-CoV-2 variant vaccine in the future?” The responses were “Yes” or “No”. Furthermore, individuals who chose “Yes” would be asked to answer the question, “Why are you willing to get vaccinated?” Response options were “the confidence in effectiveness of future vaccines”, “the trust in authorities or vaccine producers”, “the aim of protecting relatives and colleagues against the SARS-CoV-2 variant infection”, or “others”. Individuals who chose “No” would be asked, “Why are you unwilling to get vaccinated?” Response options were “sceptical about the effectiveness of the vaccine”, “lack of trust in authorities or vaccine producers”, “worried about the adverse effect of vaccine”, “There is very little chance of developing severe illness or death if I suffer from a SARS-COV-2 infection. Thus, I don’t think it is necessary to get vaccinated”, “I have little knowledge about the vaccine, and my relatives and friends advised me not to get vaccinated” or “I have a series of contraindications for vaccination”. Similarly, participants could choose one or more of the provided options.

### Statistical analysis

The basic characteristics are presented as numbers (percentages) for categorical variables and as the means (standard deviation) for continuous variables. Correspondingly, Chi-square tests or *t* tests were conducted to examine the differences. Bidirectional elimination logistic regression models were used to estimate the adjusted odds ratio (OR) along with a 95% confidence interval (95% CI) for the associations of sociodemographic characteristics, health status, and lifestyle habits with the rate of SARS-CoV-2 vaccination and the willingness to receive the SARS-CoV-2 variant vaccine. Factors included in the fully adjusted multivariable logistic regression analysis model were as follows: sex, age group, education duration, ethnicity, residence, marital status, health status, position in the group, abuse experience in the past 6 months, and history of influenza vaccination.

All statistical analyses in this study were conducted using R software version 4.0.4 (R Development Core Team, Vienna, Austria). All tests were two-sided, and a *P* value less than 0.05 was considered statistically significant. All methods were carried out in accordance with relevant guidelines.

## Results

### The basic characteristics of participants

Table 1 shows the basic characteristics of participants stratified by the status of SARS-CoV-2 vaccination. Of the 62,395 respondents (mean [SD] age: 30.83 [6.79] years), 61,712 (98.9%) received at least one dose of SARS-CoV-2 vaccine, while 683 (1.1%) never received any type of SARS-CoV-2 vaccine. Compared with unvaccinated individuals, vaccinated individuals were more likely to be younger, male, minorities, unmarried, and general workers. In addition, vaccinated individuals were more likely to come from rural areas, to have a higher rate of previous influenza vaccination and to self-report very healthy physical conditions than unvaccinated individuals. The distribution of abuse experience among vaccinated and unvaccinated individuals was comparable.

**Table 1:** The basic characteristics of participants

Characteristics	Overall (***n*** = 62,395)	Unvaccinated^**a**^ (***n*** = 683)	Vaccinated^**b**^ (***n*** = 61,712)	***P*** value
**Age, mean ± SD, years**	30.83 ± 6.79	33.29 ± 6.26	30.80 ± 6.79	< 0.001
**Age group, years**				
18–24	12,702 (20.4)	43 (6.3)	12,659 (20.5)	< 0.001
25–34	32,612 (52.3)	377 (55.2)	32,235 (52.2)	
35–44	14,966 (24.0)	225 (32.9)	14,741 (23.9)	
45–60	2115 (3.4)	38 (5.6)	2077 (3.4)	
**Sex**				
Male	45,015 (72.1)	318 (46.6)	44,697 (72.4)	< 0.001
Female	17,380 (27.9)	365 (53.4)	17,015 (27.6)	
**Education duration, years**				
1–6	240 (0.4)	5 (0.7)	235 (0.4)	< 0.001
7–9	23,710 (38.0)	168 (24.6)	23,542 (38.1)	
10–12	30,859 (49.5)	333 (48.8)	30,526 (49.5)	
13–22	7586 (12.2)	177 (25.9)	7409 (12.0)	
**Ethnicity**				
Han	54,676 (87.6)	616 (90.2)	54,060 (87.6)	0.047
Minorities	7719 (12.4)	67 (9.8)	7652 (12.4)	
**Residence**				
Urban	14,580 (23.4)	208 (30.5)	14,372 (23.3)	< 0.001
Rural	47,815 (76.6)	475 (69.5)	47,340 (76.7)	
**Marital status**				
Married	23,100 (37.0)	432 (63.3)	22,668 (36.7)	< 0.001
Unmarried	37,215 (59.6)	235 (34.4)	36,980 (59.9)	
Divorced or others	2080 (3.3)	16 (2.3)	2064 (3.3)	
**Health status**				
Very healthy	44,734 (71.7)	349 (51.1)	44,385 (71.9)	< 0.001
Well	15,842 (25.4)	239 (35.0)	15,603 (25.3)	
General or poor	1819 (2.9)	95 (13.9)	1724 (2.8)	
**Position**				
General worker	48,772 (78.2)	442 (64.7)	48,330 (78.3)	< 0.001
Line supervisor	3619 (5.8)	35 (5.1)	3584 (5.8)	
Group leader	2562 (4.1)	40 (5.9)	2522 (4.1)	
Manager	7442 (11.9)	166 (24.3)	7276 (11.8)	
**The abuse experience**				
None	55,524 (89.0)	600 (87.8)	54,924 (89.0)	0.684
Verbal abuse	4991 (8.0)	61 (8.9)	4930 (8.0)	
Physical abuse	246 (0.4)	4 (0.6)	242 (0.4)	
Both verbal and physical abuse	1634 (2.6)	18 (2.6)	1616 (2.6)	
**The history of influenza vaccination**				
Yes	21,865 (35.0)	190 (27.8)	21,675 (35.1)	< 0.001
No	40,530 (65.0)	493 (72.2)	40,037 (64.9)	

Values are presented as n (%) unless otherwise noted

*Abbreviation*: *SD* Standard deviation

^a^A person who did not receive any dose of SARS-CoV-2 vaccine was defined as a “unvaccinated individual”

^b^A person who received any dose of SARS-CoV-2 vaccine was defined as a “vaccinated individual”

### The coverage of SARS-CoV-2 vaccination and the reasons

Among 62,395 respondents, 61,081 (97.9%) received the one-dose vaccine or the second dose of the two-dose vaccine, completing the primary SARS-CoV-2 vaccination series. Furthermore, 43,716 (70.1%) had received a booster. The great majority of vaccinated individuals (94.4%) voluntarily received the SARS-CoV-2 vaccine. Other reasons for vaccination reported by the respondents ranked from the highest to lowest were as follows: being required by managers of their employers or relevant authorities (26.4%), being encouraged by relatives or friends (8.6%), and being worried about potential prejudice (4.3%). Females were more inclined than males to report voluntary vaccination (95.9 vs. 93.9%) but less inclined to report other reasons for vaccination (Table 2).

**Table 2:** The coverage of SARS-CoV-2 vaccination and the reasons

Terms	Total (***n*** = 62,395)	Male (***n*** = 45,015)	Female (***n*** = 17,380)	***P*** value
**Vaccination status**				
Unvaccinated	683 (1.1)	318 (0.7)	365 (2.1)	< 0.001
Only having received the first dose of the two-dose vaccine	631 (1.0)	488 (1.1)	143 (0.8)	
Having fully received the second dose of the two-dose vaccine	16,742 (26.8)	12,898 (28.7)	3844 (22.1)	
Having fully received the single-dose vaccine	623 (1.0)	520 (1.2)	103 (0.6)	
Having received the booster dose of SARS-CoV-2 vaccine after completing the full primary vaccination series	43,716 (70.1)	30,791 (68.4)	12,925 (74.4)	
**Reasons for the SARS-CoV-2 vaccination**^**a**^	**(*n*** **= 61,712)**	**(*n*** **= 44,697)**	**(*n*** **= 17,015)**	
I voluntarily received the vaccine	58,283 (94.4)	41,960 (93.9)	16,323 (95.9)	< 0.001
The managers of the group or relevant authorities required me to receive the vaccine	16,269 (26.4)	12,128 (27.1)	4141 (24.3)	< 0.001
My relatives or friends encouraged me to receive the vaccine	5283 (8.6)	4022 (9.0)	1261 (7.4)	< 0.001
I was worried that people around me might be prejudiced against me if I refused to receive the vaccine	2630 (4.3)	2058 (4.6)	572 (3.4)	< 0.001

Data are presented as n (%)

^a^Only vaccinated individuals answered the reasons for the SARS-CoV-2 vaccination

### The willingness to receive the SARS-CoV-2 variant vaccine and the reasons

Of the 61,712 vaccinated individuals, 60,376 (97.8%) were willing to receive the SARS-CoV-2 variant vaccine, while 1336 (2.2%) were not. Among 683 unvaccinated respondents, 588 (86.1%) were willing to receive the SARS-CoV-2 variant vaccine, while 95 (13.9%) were not. The main determinant of willingness to be vaccinated was confidence in the effectiveness of vaccines (92.8%), followed by trust in authorities or vaccine producers (68.3%) and the aim of protecting relatives and colleagues against SARS-CoV-2 variant infection (26.6%). For vaccinated participants, the top 3 reasons selected for being unwilling to be vaccinated were concern about the adverse effects of the vaccine (80.4%), being sceptical about the effectiveness of the vaccine (36.7%), and lacking trust in authorities or vaccine producers (16.8%). Among unvaccinated participants, there were a series of contraindications, including being pregnant and having underlying medical conditions, such as autoimmune disorders, nephrotic syndrome, cancer, allergies, uncontrolled epilepsy, and serious neurological disorders (85.3%); being worried about the adverse effect of the vaccine (38.9%); and being sceptical about the effectiveness of the vaccine (17.9%) (Table 3).

**Table 3:** The willingness to receive the SARS-CoV-2 variant vaccine and the reasons

Terms	Overall (***n*** = 62,395)	Vaccinated				Unvaccinated			
		Total (***n*** = 61,712)	Male (***n*** = 44,697)	Female (***n*** = 17,015)	***P*** value	Total (***n*** = 683)	Male (***n*** = 318)	Female (***n*** = 365)	***P*** value
**Being willing to be vaccinated and the reasons**	**(*n*** **= 60,964)**	**(*n*** **= 60,376)**	**(*n*** **= 43,591)**	**(*n*** **= 16,785)**		**(*n*** **= 588)**	**(*n*** **= 242)**	**(*n*** **= 346)**	
The confidence in effectiveness of future vaccines	56,597 (92.8)	56,109 (92.9)	40,321 (92.5)	15,788 (94.1)	< 0.001	488 (83.0)	189 (78.1)	299 (86.4)	0.011
The trust in authorities or vaccine producers	41,626 (68.3)	41,285 (68.4)	29,800 (68.4)	11,485 (68.4)	0.892	341 (58.0)	129 (53.3)	212 (61.3)	0.066
The aim of protecting relatives and colleagues against the SARS-CoV-2 variant infection	16,188 (26.6)	16,038 (26.6)	11,751 (27.0)	4287 (25.5)	< 0.001	150 (25.5)	63 (26.0)	87 (25.1)	0.883
Others	241 (0.4)	210 (0.3)	147 (0.3)	63 (0.4)	0.525	31 (5.3)	19 (7.9)	12 (3.5)	0.031
**Being unwilling to be vaccinated and the reasons**	**(*n*** **= 1431)**	**(*n*** **= 1336)**	**(*n*** **= 1106)**	**(*n*** **= 230)**		**(*n*** **= 95)**	**(*n*** **= 76)**	**(*n*** **= 19)**	
Skeptical about the effectiveness of the vaccine	507 (35.4)	490 (36.7)	440 (39.8)	50 (21.7)	< 0.001	17 (17.9)	14 (18.4)	3 (15.8)	1.000
Lack of trust in authorities or vaccine producers	237 (16.6)	225 (16.8)	206 (18.6)	19 (8.3)	< 0.001	12 (12.6)	9 (11.8)	3 (15.8)	0.938
Worried about the adverse effect of vaccine	1111 (77.6)	1074 (80.4)	886 (80.1)	188 (81.7)	0.634	37 (38.9)	30 (39.5)	7 (36.8)	1.000
There is very little chance of developing severe illness or death if I suffer from a SARS-COV-2 infection. Thus, I don’t think it is necessary to get vaccinated	134 (9.4)	131 (9.8)	119 (10.8)	12 (5.2)	0.014	3 (3.2)	3 (3.9)	0 (0.0)	0.883
I have little knowledge about the vaccine, and my relatives and friends advised me not to get vaccinated	132 (9.2)	126 (9.4)	106 (9.6)	20 (8.7)	0.768	5 (5.3)	4 (5.3)	1 (5.3)	1.000
I have a series of contraindications for vaccination^a^	211 (14.7)	130 (9.7)	97 (8.8)	33 (14.3)	0.013	81 (85.3)	63 (82.9)	18 (94.7)	0.347

^a^The contraindications for vaccination includes being pregnant and underlying medical condition, such as autoimmune disorder, nephrotic syndrome, cancer, allergies, uncontrolled epilepsy, and serious neurological disorders

### Multivariable logistic regression analysis: factors associated with the coverage of SARS-CoV-2 vaccination

**Fig. 1:**
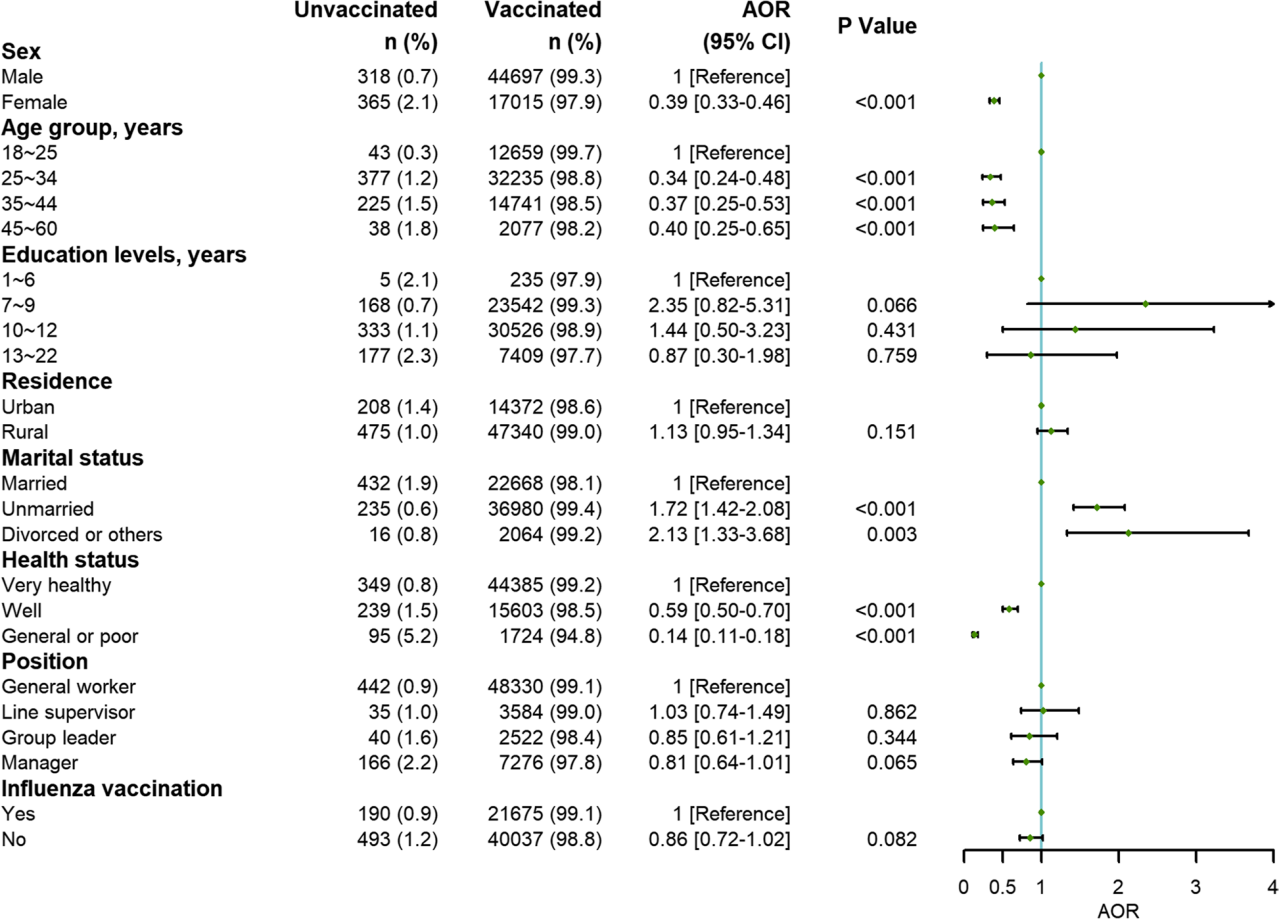
Multivariable logistic regression analysis: Factors associated with the coverage of SARS-CoV-2 vaccination. Abbreviations: OR, odds ratio; CI, confidence interval. Multivariable logistic regression adjusted for sex, age groups, education duration, residence, marital status, health status, position, and the history of influenza vaccination

Figure 1 presents the final results of the multiple logistic regression analysis that included 8 variables significantly associated with the coverage of SARS-CoV-2 vaccination. Unmarried status with a multivariable-adjusted OR of 1.72 (95% CI, 1.42-2.08), divorced status or other marital statuses (2.13; 1.33-3.68), and education duration of 7-9 years (2.35; 0.82-5.31) were associated with a higher rate of SARS-CoV-2 vaccination. Factors associated with a lower rate of SARS-CoV-2 vaccination ranked from strong to weak were as follows: general or poor health status, age of 25 or more years, female sex, manager position, and no history of influenza vaccination. The rate of vaccination was significantly lower in employees with self-reported general or poor health status (0.14; 0.11-0.18) than in those with self-reported healthy physical status.

### Multivariable logistic regression analysis: factors associated with the willingness to receive the SARS-CoV-2 variant vaccine

**Fig. 2:**
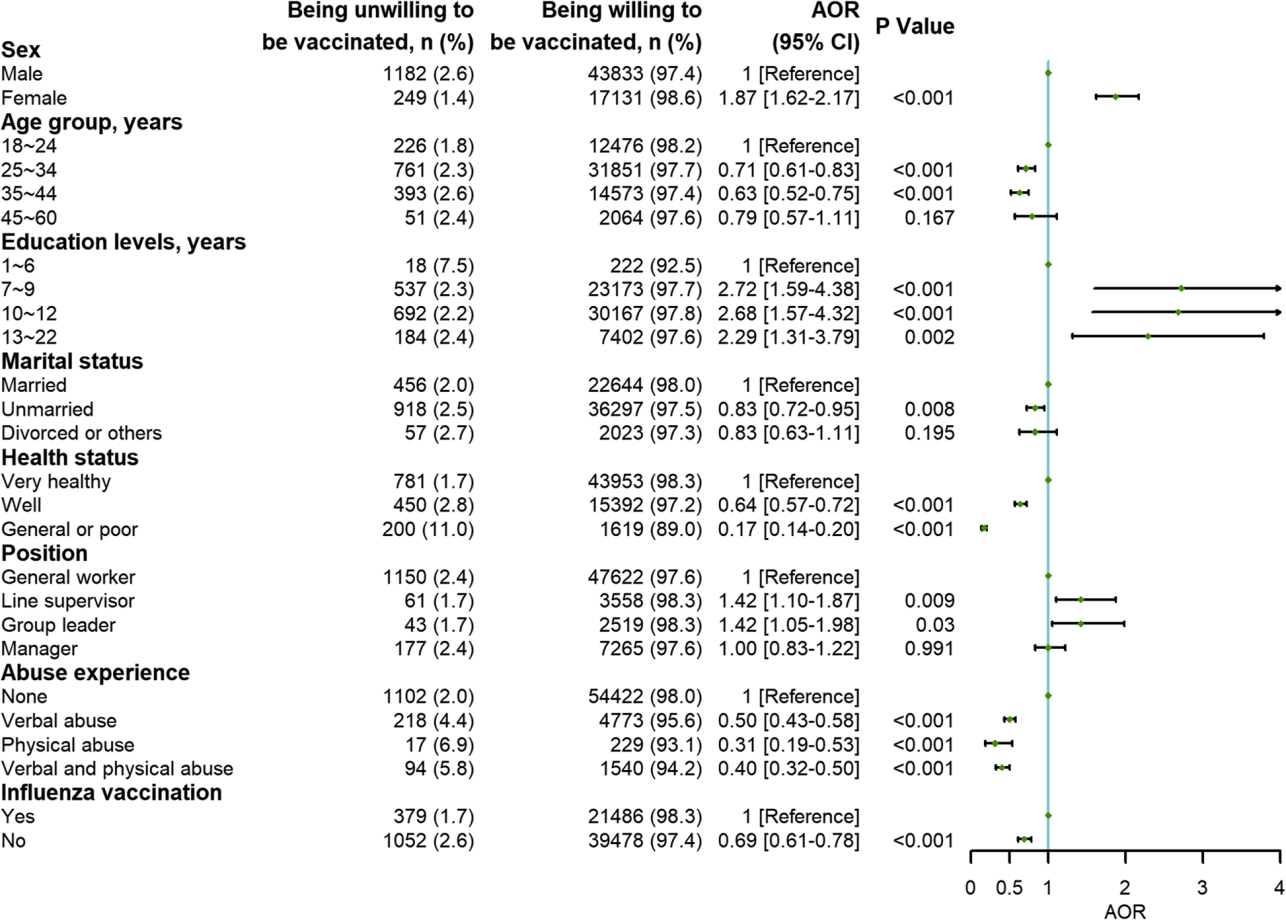
Multivariable logistic regression analysis: Factors associated with the willingness to receive the SARS-CoV-2 variant vaccine. Abbreviations: OR, odds ratio; CI, confidence interval. Multivariable logistic regression adjusted for sex, age groups, education duration, marital status, general health, position, being suffered from abuse or not, and the history of influenza vaccination

Factors positively associated with a greater willingness to receive the SARS-CoV-2 variant vaccine ranked from strong to weak were as follows: education duration of 7 years or more, female sex, and line supervisor or group leader position. For instance, participants with education durations of 7-9 years (2.72; 1.59-4.38), 10-12 years (2.68; 1.57-4.32), and 13-22 years (2.29; 1.31-3.79) were more likely to be willing to receive the vaccine than those with education durations of 1-6 years. Factors inversely associated with the willingness to receive the SARS-CoV-2 variant vaccine ranked from strong to weak were as follows: general or poor health status, abuse experience in the past 6 months, age of 25-44 years, no history of influenza vaccination, and unmarried status. Compared with participants who self-reported very healthy physical status, participants who self-reported general or poor health status (0.17; 0.14-0.20) had a lower willingness to receive the SARS-CoV-2 variant vaccine (Fig. 2).

## Discussion

Vaccination to control infectious diseases is dependent not only on vaccine efficacy and safety but also on the coverage of vaccination in the population. In this study, we observed satisfactory SARS-CoV-2 vaccination coverage of 98.9% for at least one dose and 70.1% for the booster dose, which seems to reach the requirements of herd immunity when setting the R0 of COVID-19 to 3.0. However, the estimation was based on the condition of 100% vaccine efficacy. In addition to being voluntary, the requirements of managers and encouragement of relatives or friends also played a positive role in the progress of vaccination. The results of a global survey study indicated that 48.1% of participants would receive the SARS-CoV-2 vaccine when asked by their employers [17].

A total of 60,694 respondents (97.7%) were willing to be vaccinated against SARS-CoV-2 variants in the future, which is higher than the acceptance of the SARS-CoV-2 vaccine in previous surveys [17–21]. One possible explanation is that growing evidence has indicated the efficacy of the SARS-CoV-2 vaccine in preventing severe illness and hospitalization as vaccination has progressed, which has built stronger vaccination trust among employees. In this study, we found that the influenza vaccination history positively strengthened the willingness of SARS-CoV-2 vaccination, thereby demonstrating the potential facilitative role of a reliable virus vaccination experience in building and enhancing people’s confidence in being vaccinated. A systemic review and meta-analysis [20] of 38 studies also demonstrated that people who received an influenza vaccination in the last year were more likely to accept SARS-CoV-2 vaccination, with an OR of 3.17 (95% CI, 1.84-5.46). Additionally, consistent with our findings, some emerging epidemiological evidence demonstrated that trust in government, public health authorities, scientists, and health workers significantly strengthened the willingness to take a future SARS-CoV-2 vaccination [21–23], whereas mistrust in government and public health bodies was a key barrier to vaccination [24].

It is worth noting that previous studies have also revealed that the reason why participants were unwilling to be vaccinated was scepticism about the safety or worry about the potential adverse effects of vaccines [21, 22]. Some individuals experienced fatigue, muscle pain, headache, or nausea after SASR-CoV-2 vaccination. Intensive social media coverage of serious adverse events and the spread of misleading information might also exacerbate concerns about the side effects of SASR-CoV-2 variant vaccines [21]. Additionally, persons with poorer vaccine-related knowledge were more likely to report negative future vaccination attitudes [22]. Moreover, some respondents cited low perceived risk and severity of contracting COVID-19 as an important reason for reluctance to be vaccinated [21].

More reliable information, which is based on high-quality clinical trials, on vaccine effectiveness, safety, potential and serious adverse effects should be timely and widely released by governments, health care institutions and vaccine manufacturers, and the transparency of vaccine development should be improved. Some vital steps need to be taken to diminish messages that maliciously exaggerate the adverse effects of vaccines circulating throughout social media and formulate an appropriate and moderate perceived risk of contracting COVID-19.

Similar to some previous studies [25, 26], there were significant positive associations of longer education duration with a higher rate of SARS-CoV-2 vaccination and a higher willingness to receive the SARS-CoV-2 variant vaccine. Education differences might play a crucial role in vaccination willingness among diverse populations. More efforts are required to improve the willingness to vaccinate employees at the lowest educational level.

Of note, the association of abuse experience with the willingness to get vaccinated was observed in our study. Previous studies have shown that disaster-related uncertainty, stress, fear, and economic loss have brought serious social problems, causing an increased reported frequency of abuse and domestic violence. A terrible violent experience could lead to a series of physical, psychological, and emotional consequences, delaying reaching out to health care services. People who have experienced abuse might have deeper mistrust in vaccines as well as the related institutions or organizations, which hinders the acceptance of SARS-CoV-2 vaccination. Humanistic care and psychological counselling are required for this subset of employees to strengthen vaccination willingness.

Several limitations in our study should be considered. First, given the limited resources, this survey was conducted in one large labour-intensive group, which limited the extrapolation of our findings to some extent. The employees were common blue-collar workers, such as assembly line workers, drivers, maintenance workers, storekeepers, technical engineers and managers, and valid responses covered almost all provincial administrative regions across China. Second, demographic and lifestyle factors relied on self-reporting and therefore raised the possibility of common method bias. Third, the standardized closed-ended questionnaire used in this survey led to limited information being available beyond the response options.

## Conclusion

Although the rate of SARS-CoV-2 vaccination and the willingness to receive the SARS-CoV-2 variant vaccine among employees were relatively high, some respondents were reluctant to obtain vaccinations. Adequate vaccine safety and efficacy evidence based on high-quality clinical trials should be disclosed in a timely manner by health authorities or vaccine producers to build trust among employees, especially among those with the lowest educational level, general or poor health status, abuse experience and no prior influenza vaccination history.

## Supplementary Information

**Additional file 1: eTable1.** Distribution of the Participants by Provinces or Regions (*n* = 62,395).

## Data Availability

The authors declare that the data supporting the findings of this study will be shared upon reasonable request to the corresponding author.
