# Supplementary material for: The coverage of SARS-CoV-2 vaccination and the willingness to receive the SARS-CoV-2 variant vaccine among employees in China
Source: BMC Public Health. 2023 Mar 22;23:542. doi: 10.1186/s12889-023-15294-7 (PMC10031186; doi:10.1186/s12889-023-15294-7)
Supplement: Supplementary file 1 — Additional file 1: eTable1. Distribution of the Participants by Provinces or Regions (n = 62,395). [file 12889_2023_15294_MOESM1_ESM.doc]

**eTable1. Distribution of the Participants by Provinces or Regions (n = 62,395)**

| Provinces or Regions | Number | (%) |
| --- | --- | --- |
| Hunan | 11,767 | 18.9 |
| Guangxi | 10,141 | 16.3 |
| Guangdong | 8192 | 13.1 |
| Hubei | 7264 | 11.6 |
| Henan | 5094 | 8.2 |
| Jiangxi | 4725 | 7.6 |
| Guizhou | 3239 | 5.2 |
| Sichuan | 2586 | 4.1 |
| Shaanxi | 2028 | 3.3 |
| Yunnan | 1577 | 2.5 |
| Gansu | 1010 | 1.6 |
| Chongqing | 917 | 1.5 |
| Anhui | 746 | 1.2 |
| Shandong | 415 | 0.7 |
| Hainan | 358 | 0.6 |
| Heilongjiang | 341 | 0.5 |
| Shanxi | 322 | 0.5 |
| Fujian | 320 | 0.5 |
| Hebei | 255 | 0.4 |
| Jilin | 214 | 0.3 |
| Taiwan | 185 | 0.3 |
| Liaoning | 182 | 0.3 |
| Jiangsu | 142 | 0.2 |
| Ningxia | 98 | 0.2 |
| Inner Mongolia | 93 | 0.1 |
| Zhejiang | 58 | 0.1 |
| Qinghai | 52 | 0.1 |
| Xinjiang | 42 | 0.1 |
| Tianjin | 13 | 0 |
| Beijing | 8 | 0 |
| Shanghai | 6 | 0 |
| Others regions | 2 | 0 |
| Hong Kong | 2 | 0 |
| Tibet | 1 | 0 |
